# Retraction: GNB2L1 and its O-GlcNAcylation regulates metastasis via modulating epithelial-mesenchymal transition in the chemoresistance of gastric cancer

**DOI:** 10.1371/journal.pone.0320614

**Published:** 2025-03-11

**Authors:** The PLOS One Editors

After this article [1] was published, concerns were raised about Figs 1–4.

Specifically:

- Repeated regions of similarity were noted within all panels of Figs 1B,2C and 4B.
- In Fig 3F, the left side of the SGC-7901/DDP – Control panel appears similar to the right side of the + GNB2L1 (S124A) panel.

During the editorial reassessment of this article, PLOS raised additional concerns about potential contamination or misidentification of cell lines reported in [1]. This article [1] presents the SGC-7901 cell line, and subsequently generated Adriamycin resistant SGC-7901/ADR, cisplatin resistant SGC-7901/DDP, and 5-FU resistant SGC-7901/FU, as gastric cancer cell lines. However, prior to publication of this article [1], the parent cell line, SGC-7901, was shown to be a contaminated cell line and is a potential derivative of HeLa [2]. The SGC-7901 cell line contamination was also reported in [3]. The authors did not respond to the cell line contamination concerns, and in light of these unresolved concerns, the relevance of the reported results to gastric cancer is in question.

The underlying data for the above figures of concern were not provided on request. In the absence of the original images and associated quantitative data underlying the above listed figures, these concerns remain unresolved and concerns are raised with the reliability of the associated graphical data presented in Figs 1C,2D–E,3G, and 4C–D.

In light of the above concerns, which question the reliability and validity of the reported results and conclusions, the *PLOS One* Editors retract this article.

All authors either did not respond directly or could not be reached.
